# Nationwide molecular survey of *Dirofilaria immitis* and *Dirofilaria repens* in companion dogs and cats, United States of America

**DOI:** 10.1186/s13071-022-05459-5

**Published:** 2022-10-13

**Authors:** Rachel Smith, Daniel Felipe Barrantes Murillo, Kelly Chenoweth, Subarna Barua, Patrick John Kelly, Lindsay Starkey, Byron Blagburn, Theresa Wood, Chengming Wang

**Affiliations:** 1 College of Veterinary Medicine, Auburn University, Auburn, AL USA; 2 Ross University School of Veterinary Medicine, Basseterre, Saint Kitts and Nevis; 3 Department of Pathobiology, Auburn University College of Veterinary Medicine, Auburn, AL USA

**Keywords:** *Dirofilaria immitis*, *Dirofilaria repens*, Dogs and cats, Heartworm, *Hepatozoon* molecular survey

## Abstract

**Background:**

Heartworms, *Dirofilaria immitis*, are known to be widespread in dogs and cats in the USA, but there have been no country-wide prevalence studies performed to date. There have also been no large-scale studies to determine whether the closely related species, *Dirofilaria repens*, occurs in the USA.

**Methods:**

To provide this large-scale data, we examined whole blood samples (*n* = 2334) submitted from around the USA to the Molecular Diagnostic Laboratory at Auburn University between 2016 and 2022. Quantitative PCRs for *D. immitis* (targeting 16S rRNA) and *D. repens* (targeting cytochrome c oxidase subunit 1 gene) were performed to determine the presence of *Dirofilaria* DNA. DNA sequencing was performed to confirm the results.

**Results:**

*Dirofilaria immitis* DNA was found in 6.3% (68/1080) of the dogs from 17/39 states, and 0.3% (4/1254) of the cats from 4/42 states. None of the dogs or cats were positive for *D. repens*. The average 16S rRNA copy number of *D. immitis* in the dogs was 1,809,604 in 200 µl whole blood, while only a single copy was found in each of the four *D. immitis*-positive cats. The prevalence of *D. immitis* in dogs of different ages, sexes, and breeds did not differ significantly, but the prevalence in Southern states (7.5%, 60/803) was significantly higher than in the Western (1.7%, 1/58), Midwest (3.3%, 4/120), and Northeastern states (3.1%, 3/98) (*P* < 0.05). Dogs positive for *D. immitis* were identified in each study year (2016: 4.2%, 2/48; 2017: 9.8%, 4/41; 2018: 5.1%, 8/156; 2019: 4.9%, 15/306; 2020: 9.8%, 26/265; 2021: 4.9%, 13/264). Interestingly, dogs infected with *Hepatozoon* spp. (11.8%, 37/313) were significantly more likely to also be positive for *D. immitis* than dogs without evidence of *Hepatozoon* infection (3.9%, 30/760) (*P* < 0.0001).

**Conclusions:**

To our knowledge, this is the first nationwide molecular survey of *Dirofilaria* spp. in dogs and cats in the USA, and the largest molecular survey of canine and feline dirofilariosis worldwide. Further studies are warranted to combine PCR with standard heartworm diagnostics to better understand the prevalence of *Dirofilaria* spp. and aid in determining the risks posed to dogs and cats in the USA.

**Graphical Abstract:**

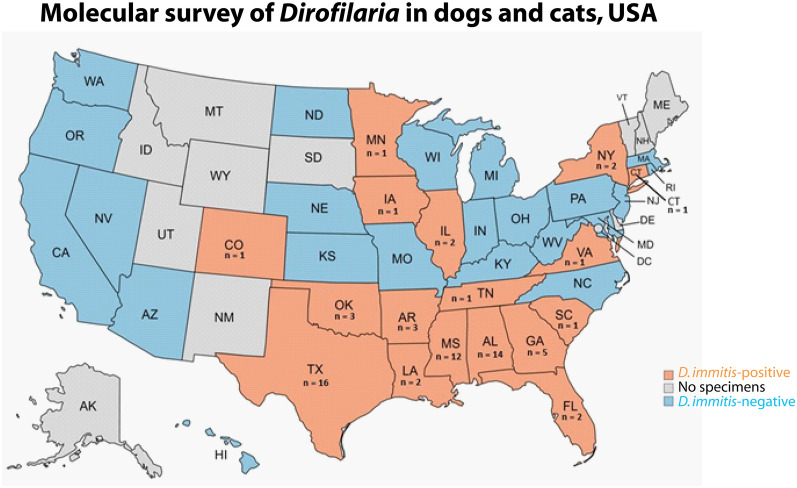

**Supplementary Information:**

The online version contains supplementary material available at 10.1186/s13071-022-05459-5.

## Background

Dirofilariosis is an arthropod-borne disease caused by filarioid nematodes belonging to the family Onchocercidae, genus *Dirofilaria*, and transmitted predominantly by mosquitoes [1]. Dirofilariosis has been reported in dogs and cats in addition to a variety of mammalian hosts, including foxes, coyotes, wolves, sea lions, harbor seals, ferrets, horses, bears, wolverines, muskrats, raccoons, bobcats, wild felids, monkeys, and red pandas [1].

The genus *Dirofilaria* includes 27 valid species and 15 species with questionable validity [1]. *Dirofilaria immitis* and *D. repens* are the most widely characterized species due to their global distribution and association with disease in companion animals and/or humans [2].

*Dirofilaria immitis* is the mosquito-borne agent of heartworm disease in dogs and cats that occurs in Africa, America, Asia, Australia, and Europe [1, 3]. Infections occur widely in the USA, and have been reported in all 50 states in dogs [6] and 48 states in cats [2, 4].

Dogs serve as the definitive host of *D. immitis*, and heartworm disease is responsible for a life-threatening systemic infection in dogs, characterized by respiratory distress, epistaxis, hemoptysis, ascites, exercise intolerance, and anorexia [1]. Bowman et al. [5] and Little et al. [6, 7] used the SNAP 3Dx^®^ or 4Dx^®^ Plus (IDEXX Laboratories, Westbrook, ME, USA) to conduct large-scale nationwide surveillance of canine dirofilariosis in the USA.

Cats are also hosts of *D. immitis*; however, infections typically result in fewer adult worms and are more difficult to detect [8]. A few prevalence studies have been conducted the in United States using antigen and/or antibody detection or parasite recovery during necropsy, and they, along with available data from the Companion Animal Parasite Council website, determined the presence of feline dirofilariosis in 48 different states in the USA [4, 9–11].

*Dirofilaria repens* is widespread across Europe and regions of Asia and Africa, and is the etiological agent of “Old World” cutaneous dirofilariosis [12]. No nationwide studies have been conducted to explore the prevalence of *D. repens* in the dogs and cats of the USA.

While the identification of microfilariae in blood specimens by microscopy can be used to identify *D. immitis* infection [2], antigen detection is now considered the gold standard due to its sensitivity and specificity [2]. The highly sensitive and specific enzyme immunosorbent assay (ELISA) and immunochromatography-based diagnostic techniques for detecting circulating antigens have been widely used by veterinarians since 1985 [13]. However, antigen detection-based assays are not infallible as a low level of cross reactions have been reported to the circulating antigens of a few parasites [13–19]. In comparison, polymerase chain reaction (PCR)-based diagnostic methods focus on the amplification and sequencing of DNA and demonstrate high specificity to distinguish between other medically relevant species such as *D. repens.* Molecular detection of *D. immitis* has been proved as a highly sensitive, specific and reliable method for the detection of small amounts of genomic DNA in dog blood and even mosquito vectors [19–21].

To add to the existing antigen and antibody prevalence data on *D. immitis* infections in dogs and cats in the USA, we carried out a large-scale molecular survey to provide large-scale molecular data on the presence of *D. immitis* and *D. repens* in the blood of dogs and cats in the USA.

## Methods

### Whole blood samples

Samples in this study included whole blood samples in EDTA from dogs (*n* = 1080) submitted to the Molecular Diagnostic Laboratory at Auburn University between 2016 to 2021 for diagnosis of babesiosis (*n* = 4), bartonellosis (*n* = 2), dirofilariosis (*n* = 1), and hepatozoonosis (*n* = 1073), and blood samples from cats (*n* = 1254) for the diagnosis of feline infectious peritonitis between 2016 to 2022. Fluorescence resonance energy transfer (FRET) PCRs were performed to detect nucleic acids of *Hepatozoon* spp., *D. immitis*, *Babesia* spp., *Bartonella* spp. and feline coronavirus.

Samples from dogs were submitted from 39 states of the USA. States with no submitted samples included Alaska, Delaware, Idaho, Maine, Montana, New Hampshire, New Mexico, South Dakota, Utah, Vermont, and Wyoming (Table 1).

**Table 1:** Molecular prevalence of *D. immitis* in whole blood samples of dogs by state

Origin state of the sample	Total samples	Positive samples	Percent positivity
Alabama	151	14	9.3%
Arizona	2	0	0.0%
Arkansas	15	3	20.0%
California	25	0	0.0%
Colorado	19	1	5.3%
Connecticut	6	1	16.7%
Florida	38	2	5.3%
Georgia	78	5	6.4%
Hawaii	1	0	0.0%
Illinois	19	2	10.5%
Indiana	15	0	0.0%
Iowa	8	1	12.5%
Kansas	27	0	0.0%
Kentucky	4	0	0.0%
Louisiana	23	2	8.7%
Maryland	2	0	0.0%
Massachusetts	26	0	0.0%
Michigan	6	0	0.0%
Minnesota	13	1	7.7%
Mississippi	108	12	11.1%
Missouri	6	0	0.0%
Nebraska	1	0	0.0%
Nevada	4	0	0.0%
New Jersey	4	0	0.0%
New York	48	2	4.2%
North Carolina	8	0	0.0%
North Dakota	1	0	0.0%
Ohio	6	0	0.0%
Oklahoma	123	3	2.4%
Oregon	3	0	0.0%
Pennsylvania	12	0	0.0%
Rhode Island	2	0	0.0%
South Carolina	11	1	9.1%
Tennessee	12	1	8.3%
Texas	218	16	7.3%
Virginia	11	1	9.1%
Washington	5	0	0.0%
West Virginia	1	0	0.0%
Wisconsin	18	0	0.0%
Total	1080	68	6.3%

Feline blood samples were submitted from 42 states of the USA in this study. States with no submitted samples were Delaware, Hawaii, Montana, North Dakota, South Dakota, Vermont, West Virginia, and Wyoming.

The blood samples were shipped at ambient temperature to Auburn University, and DNA was extracted from 800 µl aliquots using the High-Pure PCR Template Preparation Kit (Roche Molecular Biochemicals, Indianapolis, IN, USA) as published [22]. The DNA was eluted in 100 µl elution buffer, and the 20 µl volume of each sample remaining after other PCR assays had been performed was preserved at −80 ºC until use for detecting *D. immitis* and *D. repens* in this study.

### Detection of *D. immitis* and* D. repens* DNA by PCRs

Quantitative PCR (qPCR) [20] was used to detect DNA of *D. immitis* and *D. repens* in this study. The *D. immitis* PCR targets a 16S rRNA gene using the primers (ImmF: 5′-CTA TAT GTT ACC TTA ATT GG-3′; ImmR: 5′-CTT AAC CAT TAT CTT AGA TCA G-3′) and the probe (ImmT: 5′-ROX-GTA GCT AGT AAG TTT ACC TTG-BHQ2-3′; ROX = 6-carboxy-X-rhodamine, BHQ2 = black hole quencher 2; the amplicon size is 162 bp). For the *D. repens* PCR, a cytochrome c oxidase subunit 1 gene fragment was amplified using the primers (RepF: 5′-GAG ATG GCG TTT CCT CGT G-3′; RepR: 5′-GAC CAT CAA CAC TTA AAG-3′) and the probe (RepT: 5′-JOE-GTT GCT TTG TTA ATG GTT TAT C-BHQ1-3′; JOE = 6-carboxy-4′,5′-dichloro-2′,7′-dimethoxyfluorescein, BHQ1 = black hole quencher 1; the amplicon size is 139 bp) [20].

PCR was performed on a Roche LightCycler 480 II thermocycler (Roche Diagnostics GmbH, Mannheim, Germany). In brief, 10 µl of the extracted DNA was added to a 10 µl reaction mixture containing 5 × PCR FRET buffer, 400 µM dNTP (Roche Diagnostics GmbH), 0.34 units of Platinum Taq DNA Polymerase (Invitrogen, Carlsbad, CA, USA), 1 µM of each forward and reverse primer (Integrated DNA Technologies, Coralville, IA, USA), and a final volume of molecular grade nuclease-free water.

The product of *D. immitis* PCR on DNA extracted from *D. immitis*-positive samples (kindly provided by the Laboratory of Veterinary Parasitology at Auburn University College of Veterinary Medicine) was used as positive control and quantitative standards. After using the molecular mass of the rRNA gene to calculate the molarity of the solution, dilutions of PCR products were made to give solutions containing 10,000, 1000, 100, 10, and 1 gene copies per reaction.

The plasmid containing the amplicon region was ordered from IDT (Integrated DNA Technologies, Coralville, IA, USA) to use as positive control and quantitative standards in the *D. repens* PCR.

The products of *Dirofilaria*-positive PCRs were sent to ELIM Biopharmaceuticals (Hayward, CA, USA) for Sanger DNA sequencing using upstream and downstream primers.

## Statistical analysis

All statistical analyses were performed with the Statistica 7.0 software package (StatSoft, Inc., Tulsa, OK, USA). A Chi-square test was performed to compare the positivity of *D. immitis* DNA between dogs and cats, and in dogs from different geographical regions, between females and males, and those positive or negative for *Hepatozoon* spp. A difference at *P* ≤ 0.05 was considered significant.

## Results

The *D. immitis* and *D. repens* PCRs used in this study were found to be specific and sensitive, in both cases being able to detect one copy of the target gene in 20 µl of reaction mixture. While none of the dog or cat samples were positive for *D. repens* DNA, the *D. immitis* qPCR identified 81 positive dogs and four positive cats. DNA sequencing of the qPCR products using upstream and downstream primers confirmed *D. immitis* in 68 of the 81 dog samples and all four positive cat samples. The 13 qPCR-positive samples that did not provide clean DNA sequences were not included in further analysis.

Significantly more dogs were positive for *D. immitis* (6.3%; 68/1080) than cats (0.3%; 4/1254) (*P* < 0.0001). The average 16S rRNA copy number in the dogs was 1,809,604 (±2,455,094 standard deviation) in 200 µl whole blood from which DNA was extracted, while a single copy was found in each of the four *D. immitis*-positive cats.

**Fig. 1:**
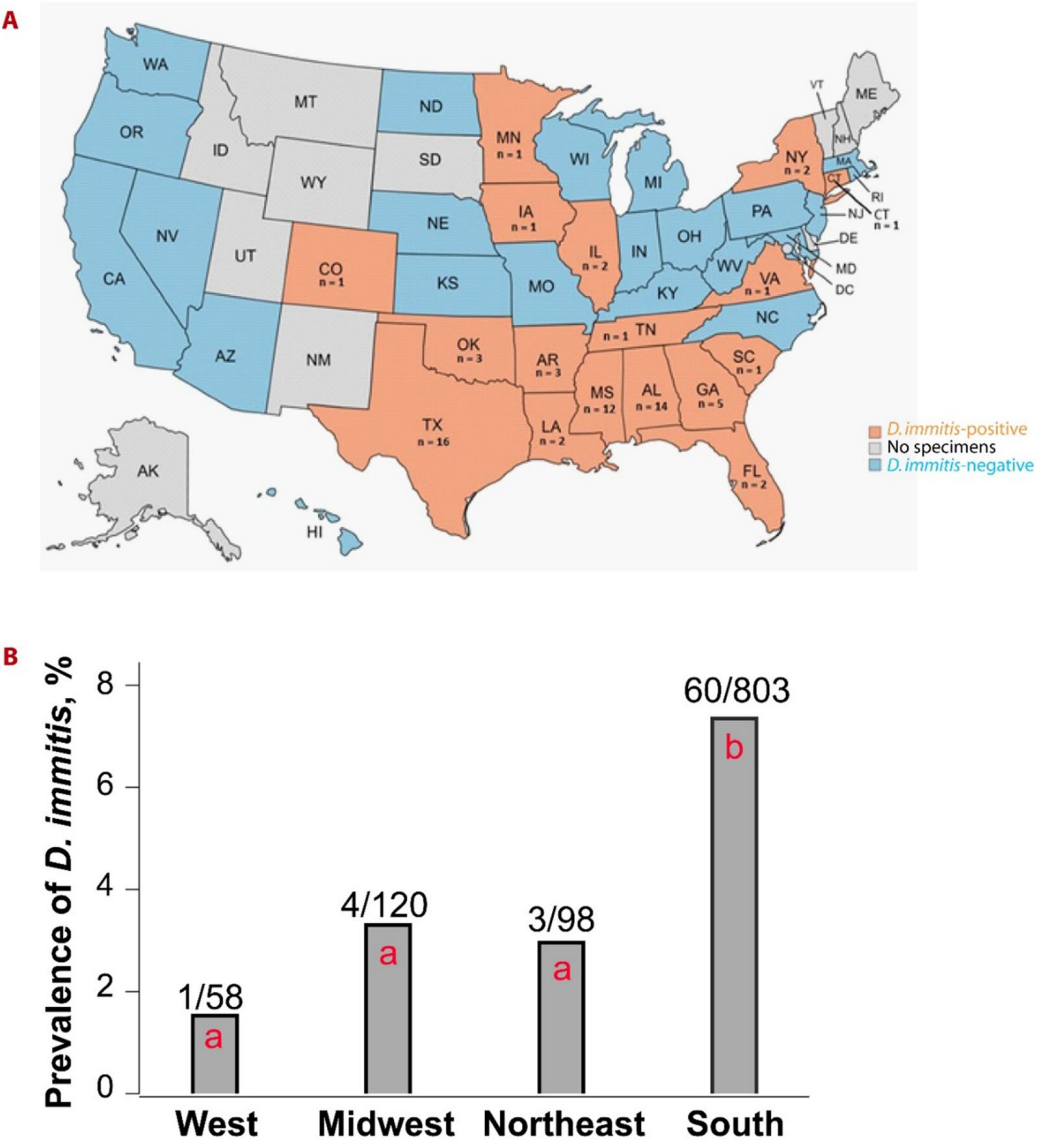
*Dirofilaria immitis* prevalence in canines by state and geographical region. **a** Among 39 states with specimens available for this study, *D. immitis* DNA was identified in canine blood samples from 17 states; **b** *D. immitis* prevalence in dogs from the South region was 7.5% (68/803), being significantly higher than in the West (1.7%, 1/58), Midwest (3.3%, 4/120), and Northeast (3.1%, 3/98) (*P* < 0.5; Chi-square test). Different letters (a, b) denote significant difference. Only one canine sample was submitted from the Pacific region, which was not included in the comparison. The definition of different regions can be found at www. chrome-extension://efaidnbmnnnibpcajpcglclefindmkaj/https://www2.census.gov/geo/pdfs/maps-data/maps/reference/us_regdiv.pdf

Dogs that were positive for *D. immitis* were found in 17 of the 39 (43.6%) states from which samples were available (Table 1, Additional file 1: Table S1, Fig. 1a). The prevalence in dogs from the southern USA (7.5%, 60/803) was significantly higher than in the West (1/58, 1.7%), Midwest (4/120, 3.3%), or Northeast (3/98, 3.1%) (*P* < 0.05; Chi-square test) (Fig. 1b). The average age of the positive dogs was 4.63 (±3.18 standard deviation) years, with the youngest being 10 months and the oldest 13 years.

Most infected dogs were reported on the sample submission form as mixed breeds (*n* = 38), with the remainder reported as pure breeds comprising German Shepherds (7), Pit Bulls (4), Beagles (3), Great Danes (2), and a single Border Collie, Boxer, Catahoula, Chihuahua, Coton de Tuléar, Jack Russell Terrier, Pomeranian, Scottish Terrier, and Shiba Inu (Additional file 1: Table S1). No significant differences were found in the prevalence of *D. immitis* in dogs of different ages, sexes, and breeds.

Dogs positive for *D. immitis* were identified in each study year—4.2% in 2016 (2/48), 9.8% in 2017 (4/41), 5.1% in 2018 (8/156), 4.9% in 2019 (15/306), 9.8% in 2020 (26/265), and 4.9% in 2021 (13/264).

While none of the samples initially submitted for *Bartonella* spp. (2) and *Dirofilaria immitis* (1) testing were positive, one of the samples submitted for *Babesia* spp. testing was positive (25.0%; 1/4), as were 67 of those submitted for *Hepatozoon* spp. testing (6.3%; 67/1073). Dogs positive for *Hepatozoon* spp. (11.8%, 37/313) were significantly more likely to be concurrently positive for *D. immitis* than dogs that were negative for *Hepatozoon* spp. (3.9%, 30/760) (*P* < 0.0001; Chi-square test).

*Dirofilaria immitis* DNA was identified in only four cats (0.3%; 4/1254). All positive cats were female domestic shorthairs that were 1 (from Mississippi), 2 (Florida), 7 (Georgia), and 11 (New York) years old.

## Discussion

*Dirofilaria immitis* infections in dogs are a persistent and serious problem in the USA, with over 200,000 dogs testing positive for heartworm in 2021 alone [4]. Infections can also occur in cats, and generally produce different clinical signs from those seen in dogs and may be life-threatening, with only support treatments available. Despite the potentially serious nature of the disease in cats, there is little information regarding the prevalence and risk factors for feline heartworm disease. *Dirofilaria repens*, the causative agent of ocular and subcutaneous dirofilariosis [2], is found almost exclusively in the Old World and is considered absent in the USA [23], although substantive surveillance data are lacking. To further understand the geographical distribution of *Dirofilaria* spp. in the USA and the risk factors that may contribute to the persistent burden of heartworm disease in companion animals, we used highly sensitive and specific PCRs to carry out a molecular survey for *D. immitis* and *D. repens* in dogs and cats from around the country.

The prevalence of *D. immitis* infections identified in this study (6.3%, 68/1080) was considerably higher than the 1.4% (2001‒2007: 43,500/3,182,301), 1.3% (2010‒2017: 142,426/10,734,132), and 1.4% (2013‒2019: 651,347/47,254,819) reported in large-scale nationwide antigen-based surveillance studies [5–7]. Antigen-based detection methods have become considered the gold standard diagnostic test due to their sensitivity and specificity [1, 2]. More recently developed PCR-based diagnostic methods are not suitable for use in general veterinary practice but are considerably more sensitive and specific than the antigen-based tests [19–21]. It is also important to note that immune complex dissociation (ICD) treatments of the plasma and sera have been found to increase the detection of *D. immitis* antigen [24, 25]. The prevalence of *Dirofilaria* spp. in previously reported nationwide surveillance studies was not determined with these treatments, and this might explain why their prevalence was lower than ours. It is also possible that the differences in prevalence between our molecular-based survey and that of the previously published antigen-based reports might be due to differences in sample sizes and the health and environmental conditions of the tested animals [5–7].

Domestic dogs serve as the definitive host for *D. immitis* and become infected when third-stage larvae are deposited near the bite wound following feeding by an infected *Aedes albopictus*, *Culex pipiens*, *Anopheles punctipennis*, or *Mansonia uniformis* mosquito [5, 7]. Climate appears to be a critical factor in the distribution of infections, with the prevalence of *D. immitis* known to be higher in warmer climates. We found significantly more infected dogs in the warmer southern states (7.5%, 60/803) as compared with the cooler West (1/58; 1.7%), Midwest (4/120; 3.3%), and Northeast (3/98; 3.1%) USA. Our findings are consistent with previously published data from antigen-based studies of *D. immitis* infections in dogs [5–7]. Little et al. [6] reported that *D. immitis* infection in canines was highest in the southeastern USA, with a 2.6% prevalence, compared to the West, Midwest, and Northeast, with prevalence of 0.7%, 0.8%, and 0.5%, respectively.

Interestingly, we found that dogs PCR-positive for *Hepatozoon* spp. (11.8%, 37/313) were significantly more likely to be concurrently positive for *D. immitis* DNA than dogs that were negative for *Hepatozoon* spp. (3.9%, 30/760) (*P* < 0.0001). Similarly, co-infections with other vector-borne agents have been shown as risk factors for heartworm disease, mainly *Anaplasma* spp., *Babesia* spp., *Borrelia burgdorferi*, *Ehrlichia* spp., and *Trypanosoma cruzi* [26–29]. While infections with other vector-borne pathogens may downregulate the host’s immune system and predispose to co-infections, it seems more likely that living conditions and environmental factors play an important role. Dogs that live outdoors are more likely to be exposed to a variety of vector species, not just mosquitos, thus increasing the opportunity for pathogen transmission and co-infections with other vector-borne agents [29].

The prevalence of *D. immitis*-positive samples from cats in our study, not unsurprisingly, was low. The markedly lower prevalence in positive feline samples compared with dogs is directly related to the pathophysiology of infections in cats. Overall, dirofilariosis in cats is characterized by a much lower burden of adult and immature worms than is seen in dogs, and this is accompanied by lower microfilariae and antigen concentrations in the blood [2]. Further, adult worms have shorter life spans in cats, only 2–4 years [30].

While *D. repens* is very common in the Old World, we found no evidence of infections in the USA, supporting previous assertions that ocular and subcutaneous dirofilariosis [2] are considered exotic in the USA [23]. It is a somewhat surprising finding, as the major vector mosquitos of *D. repens* are not uncommon in the USA, mainly *Culex pipiens pipiens*, *Ae. albopictus*, and *Aedes vexans* [31]. Further studies are indicated to continue monitoring the situation.

Our study has several limitations. Firstly, the samples were not randomly selected, as the vast majority of the canine samples (99.4%; 1072/1080) were submitted for the molecular diagnosis of *Hepatozoon* spp. and all the feline samples for the PCR diagnosis of feline infectious peritonitis. Secondly, we had no epidemiological data which could have influenced infections such as whether animals were indoors or outdoors, the use of dirofilariosis prophylaxis treatments, travel history, and the clinical signs present with their history. Furthermore, PCR detection of DNA in whole blood relies mostly or entirely on the presence of circulating microfilariae of the *Dirofilaria* spp. A number of factors may play a role in the limited presence of microfilariae in dogs and cats, such as intermittent use of preventive drugs that can impact microfilaria numbers; infections that comprise sexually immature worms, single-sex infections, or too few worms to sexually reproduce; or targeting of microfilaria by the host’s immune system. Lastly, due to the volume of submitted samples, traditional heartworm diagnostics could not be pursued for full comparison. A more precise description of the prevalence of *D. immitis* infections across the USA would require further study addressing these limitations.

Most heartworm prevalence studies conducted in the USA have opted for antigen detection potentially with the addition of microfilaria visualization. Antigen detection is considered the gold standard due to its sensitivity and specificity [2]. However, quantitative PCR might be considered as a practical tool to detect *Dirofilaria* spp. microfilariae due to its exquisite sensitivity and specificity as well. Ideally, microscopy using the Knott’s tests, antigen test, antibody detection, and PCR will be compared for their diagnostic accuracy in the animal model experimentally infected with *D. immitis*.

## Conclusions

To the best of our knowledge, this is the largest molecular survey of canine and feline dirofilariosis worldwide, and the first nationwide molecular survey of *Dirofilaria* spp. in companion animals in the USA. *Dirofilaria immitis* DNA was found in 6.3% (68/1080) of the dogs and 0.3% (4/1254) of the cats, while none of the dogs or cats were positive for *D. repens*. Dogs infected with *Hepatozoon* were significantly more likely to be positive for *D. immitis* co-infection than non-*Hepatozoon*-infected dogs. Further studies are needed with samples from dogs and cats with more precise histories of risk factors and the use of heartworm prophylaxis. These samples would be tested by both PCR and standard heartworm diagnostics, to better understand the occurrence of *Dirofilaria* spp. and to more precisely establish risks of infection.

## Supplementary Information

**Additional file 1: Table S1***Dirofilaria immitis*-positive samples in dogs identified in this study.

## Data Availability

The data sets generated during the current study are available from the corresponding author on reasonable request.

## Abbreviations

*ROX*: 6-Carboxy-X-rhodamine
*BHQ2*: Black hole quencher 2
*JOE*: 6-Carboxy-4′,5′-dichloro-2′,7′-dimethoxyfluorescein
*BHQ1*: Black hole quencher 1
