# Supplementary material for: Nationwide molecular survey of Dirofilaria immitis and Dirofilaria repens in companion dogs and cats, United States of America
Source: Parasit Vectors. 2022 Oct 13;15:367. doi: 10.1186/s13071-022-05459-5 (PMC9559157; doi:10.1186/s13071-022-05459-5)
Supplement: Supplementary file 1 — Additional file 1: Table S1Dirofilaria immitis-positive samples in dogs identified in this study. [file 13071_2022_5459_MOESM1_ESM.docx]

**Additional file 1: Table S1.** *Dirofilaria immitis*-positive samples in dogs identified in this study

| **Date** | **Age, year** | **Sex** | **Breed** | **Copy number**** | **State** |
| --- | --- | --- | --- | --- | --- |
| 2/19/2016 | 4 | Male | Mixed Breed | 7.02 | NY |
| 10/4/2016 | N/A***** | Male | Mixed Breed | 6.90 | NY |
| 4/28/2017 | N/A | Female | Great Dane | 3.38 | TX |
| 7/12/2017 | 7 | Female | Pit Bull | 6.85 | TX |
| 8/7/2017 | 1.5 | Female | Mixed Breed | 6.81 | TX |
| 8/22/2017 | N/A | Male | Beagle | 6.78 | AL |
| 3/7/2018 | 4 | Male | German Shepherd | 6.77 | LA |
| 7/24/2018 | 5 | Female | Mixed Breed | 6.76 | MS |
| 7/30/2018 | 5 | Male | N/A | 6.75 | AL |
| 8/7/2018 | 14 | Male | Chihuahua | 3.37 | MS |
| 9/10/2018 | 1.5 | Female | Mixed Breed | 0.79 | AL |
| 10/31/2018 | 4 | Male | German Shepherd | 6.72 | OK |
| 11/8/2018 | 3.17 | Female | Jack Russell Terrier | 3.68 | LA |
| 11/15/2018 | 5 | Female | Mixed Breed | 6.69 | TX |
| 1/7/2019 | 6 | Male | German Shepherd | 3.35 | SC |
| 2/6/2019 | 1 | Female | Pit Bull | 2.95 | AL |
| 2/12/2019 | 10 | Male | Coton De Tulear | 3.58 | FL |
| 3/22/2019 | 4 | Male | Lab Mix | 0.21 | TX |
| 4/10/2019 | 10 | Female | Mixed Breed | 3.56 | FL |
| 4/19/2019 | 2 | Male | Mixed Breed | 2.73 | AL |
| 5/31/2019 | 2 | Female | Terrier Mix | 2.48 | MS |
| 8/9/2019 | 3 | Female | Pit Bull Mix | 2.47 | GA |
| 8/30/2019 | 2.4 | Female | Golden Retriever Mix | 4.61 | CT |
| 10/14/2019 | 1 | Male | Mixed Breed | 6.69 | AR |
| 11/19/2019 | 2 | Male | German Shepherd | 2.08 | GA |
| 11/21/2019 | 4 | Female | Beagle Mix | 6.66 | AR |
| 11/21/2019 | 5 | Female | Mixed Breed | 6.61 | MS |
| 12/27/2019 | N/A | N/A | N/A | 6.60 | TX |
| 12/30/2019 | 2 | Female | Beagle | 2.04 | MS |
| 1/9/2020 | N/A | N/A | N/A | 6.58 | TX |
| 1/31/2020 | 13 | Male | Catahoula Leopard Dog | 6.55 | TX |
| 2/3/2020 | 3 | Female | Hound Mix | 6.55 | AL |
| 3/11/2020 | 8.5 | Female | Pomeranian | 1.87 | TX |
| 3/18/2020 | 0.83 | Female | Terrier Mix | 6.53 | MN |
| 4/20/2020 | 8 | Male | Pit Bull | 6.46 | TX |
| 4/27/2020 | 10 | Male | Scottish Terrier | 1.61 | AL |
| 4/30/2020 | 2 | Male | N/A | 4.48 | MS |
| 5/11/2020 | 1.83 | Male | Great Dane | 6.45 | MS |
| 5/21/2020 | 2 | Male | Mixed Breed | 6.39 | AL |
| 6/2/2020 | 4.25 | Male | Labrador Retriever | 6.32 | AL |
| 6/4/2020 | 3.5 | Female | Labrador Retriever | 6.22 | MS |
| 6/24/2020 | 3 | Male | Pit Bull Mix | 6.20 | TX |
| 8/21/2020 | 5 | Female | Labrador Retriever | 6.17 | OK |
| 8/26/2020 | 4 | Female | German Shepherd | 6.15 | MS |
| 8/27/2020 | 5 | Male | Beagle | 1.56 | IL |
| 9/16/2020 | 5 | Male | Border Collie | 1.40 | AL |
| 9/28/2020 | 3 | Male | Lab Mix | 6.15 | TX |
| 10/19/2020 | 1 | Female | Golden Retriever Mix | 6.14 | TX |
| 10/21/2020 | 3 | Male | Spaniel Mix | 6.08 | GA |
| 11/6/2020 | 5 | Male | Lab Mix | 6.02 | IL |
| 11/12/2020 | 4 | Female | Labrador Retriever | 5.87 | TN |
| 11/12/2020 | 11 | Female | Terrier Mix | 1.23 | VA |
| 11/24/2020 | 2 | Female | Beagle Mix | 5.84 | TX |
| 12/2/2020 | 4 | Male | Mixed Breed | 1.06 | AR |
| 12/9/2020 | 5 | Female | Labrador Retriever | 5.79 | TX |
| 1/27/2021 | N/A | Male | Mixed Breed | 0.80 | AL |
| 2/8/2021 | 2 | Male | Terrier Mix | 5.57 | CO |
| 4/8/2021 | 10 | Female | Shiba Inu | 5.54 | OK |
| 4/28/2021 | 6 | Male | Mixed Breed | 5.52 | GA |
| 5/17/2021 | N/A | N/A | N/A | 5.40 | MS |
| 5/25/2021 | 1 | Male | Boxer | 5.37 | AL |
| 6/11/2021 | 13 | Female | Mixed Breed | 5.31 | TX |
| 7/8/2021 | 4 | Male | Lab Mix | 4.99 | AL |
| 9/13/2021 | 5 | Female | German Shepherd | 4.92 | MS |
| 9/24/2021 | 4.5 | Female | Pit Bull | 4.90 | AL |
| 9/24/2021 | 4 | Male | German Shepherd | 4.80 | IA |
| 10/7/2021 | 6 | Female | Mixed Breed | 4.78 | GA |
| 10/13/2021 | 2 | Male | Mixed Breed | 4.64 | MS |

* N/A indicates the age or gender of the dog was not available;

** The log _10_ copy number of *D. immitis* per 200 µL whole blood was determined by *D. immitis* quantitative PCR.
